# Acute Brachial Artery Occlusion in an Elderly Patient With Acute Myocardial Ischemia

**DOI:** 10.7759/cureus.1700

**Published:** 2017-09-19

**Authors:** Gabriel O Ologun, Christian Bohan, Tiffany Lau, Mohammad Sultany, Andrew Trecartin, Zachary Wolfe, Silviu Marica, Lawrence Sampson, Umashankar Ballehaninna

**Affiliations:** 1 General Surgery, Guthrie Clinic/Robert Packer Hospital; 2 Medical Student, Geisinger Commonwealth School of Medicine; 3 Internal Medicine, Guthrie Clinic/Robert Packer Hospital; 4 Vascular Surgery, Guthrie Clinic/Robert Packer Hospital

**Keywords:** hand ischemia, brachial artery embolism, arterial occlusion upper extremity, arrhythmia

## Abstract

We present a case of left upper extremity paresis secondary to acute brachial artery occlusion in an elderly female with active non-ST segment elevation myocardial ischemia (NSTEMI) in the setting of paroxysmal atrial fibrillation. The patient was initially suspected to have a cerebrovascular attack (CVA); however, computed tomography (CT) head was negative for acute stroke. The diagnosis was confirmed by computed tomographic angiography (CTA) of the upper extremity, confirming the diagnosis of acute left brachial artery occlusion. In evaluating a patient with concern for acute stroke with atypical presentation, it is essential to obtain a complete history and perform a rapid and thorough examination. Acute limb ischemia (ALI) should be considered in the differential diagnosis of CVA with atypical presentation.

## Introduction

The clinical burden of peripheral arterial events is substantial, with high case fatality, poor functional outcome, and a high rate of emergency and subsequent vascular intervention to prevent death and limb loss [1]. We present a case of left upper extremity paresis secondary to acute brachial artery occlusion in an elderly female with active non-ST segment elevation myocardial ischemia (NSTEMI) in the setting of paroxysmal atrial fibrillation. The patient was initially suspected to have a cerebrovascular attack (CVA); however, computed tomography (CT) head was negative for acute stroke. The presence of skin mottling, coolness of the arm, pallor, decreased sensation, and pulseless led to a further investigation with a computed tomographic angiography (CTA) of the upper extremity, confirming the diagnosis of acute left brachial artery occlusion. Acute limb ischemia should be considered in the differential diagnosis of acute stroke with atypical presentation.

## Case presentation

The patient is an 83-year-old female who presented to an outside rural hospital emergency department with a complaint of nausea and chest discomfort of one-day duration. Her medical history included hypertension, hyperlipidemia, carotid artery stenosis, traumatic subarachnoid hemorrhage, transient ischemic attack, and paroxysmal atrial fibrillation. She is a nonsmoker. On exam, she was normocardic with a regular rhythm. The rest of her vital signs were normal. Laboratory investigation revealed white blood cell count 6.9 K/uL, hemoglobin 14.6 g/dL, platelet count 170 K/uL, lipase 21, and an abnormal troponin level 0.180 ng/mL. An electrocardiogram (ECG) obtained reveal normal sinus rhythm, pulse rate 60. The emergency department physician diagnosed non-ST elevation myocardial ischemia (NSTEMI). Her chest discomfort improved with administration of nitroglycerin and morphine; anticoagulation with heparin drip was initiated and she was transferred to our facility. Her home medications include baby aspirin, metoprolol, and ramipril.

On arrival, she complained of acute onset left upper extremity weakness. A stroke alert was initiated. The patient’s systolic blood pressure was 180 mmHg and the ECG demonstrated atrial fibrillation at 90 beats per minute. On neurologic examination, the left upper extremity strength revealed no effort against gravity with some preserved strength in wrist and finger extension. Diminished sensation to gross touch on the left forearm compared to the right. Findings from the remainder of the neurological examination, including speech and language, cranial nerves, coordination, and left lower extremity strength and sensation, were normal. A computed tomography scan of the brain showed no gross evidence of intracranial hemorrhage. There was hypodensity representing age-indeterminate infarct within the left occipital lobe and age-indeterminate periventricular and subcortical small vessel ischemic changes.

**Figure 1:**
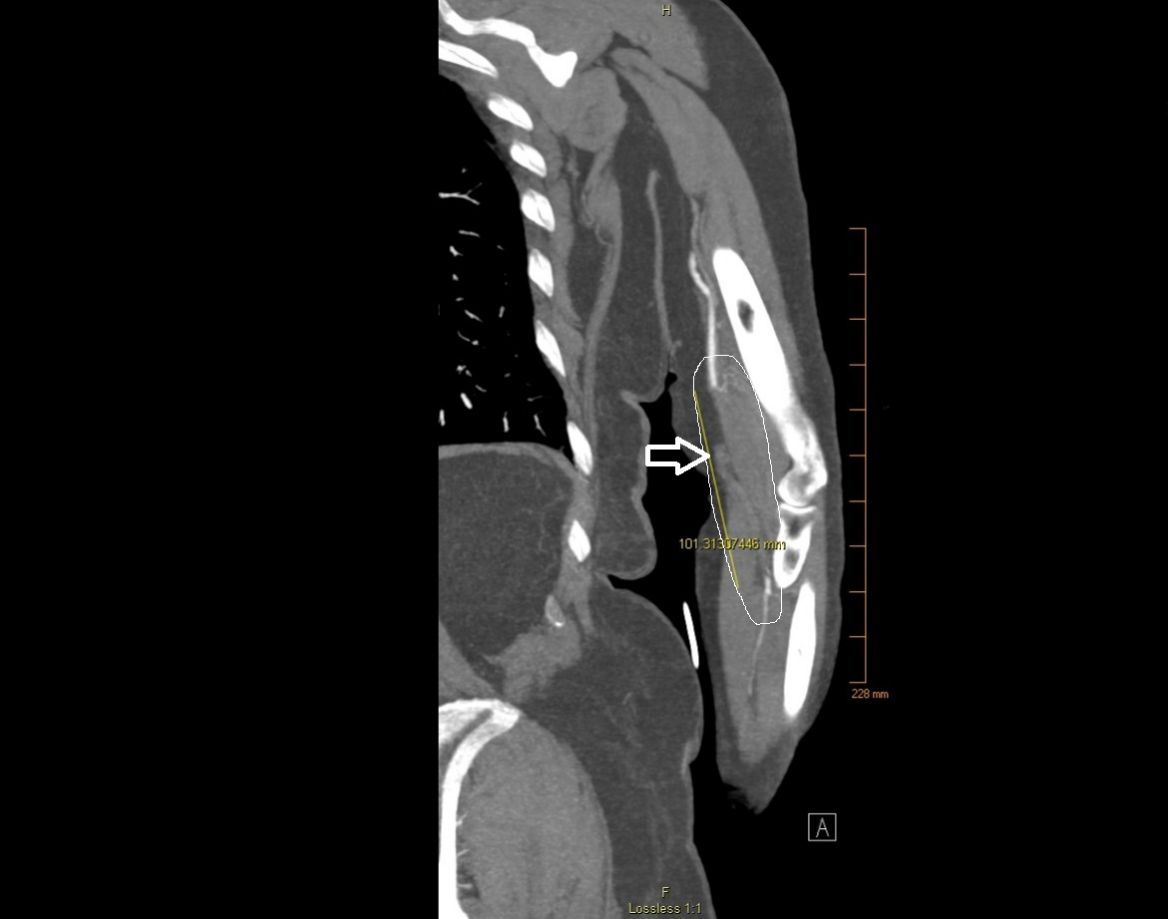
CTA of left upper extremity: Coronal view demonstrating complete occlusion of the distal brachial artery measuring 10 cm in length (outlined and arrow). There is diminished flow in the radial and ulnar arteries. CTA: Computed tomographic angiography

Given that weakness and mottling were prominent, palpation of the left upper extremity for pulses was done. The left brachial, radial, and ulnar pulses were not palpable, and the left upper extremity was colder than the right upper extremity. There were also no appreciable Doppler signals. A CTA of the left upper extremity was performed, which revealed left distal brachial artery occlusion (Figure 1). Vascular surgery was consulted. Intravenous heparin drip was resumed and the patient was sent to the operating room for emergent left upper extremity exploration and embolectomy of the left brachial artery.

Postoperatively, a transthoracic echocardiogram obtained was negative for an intracardiac thrombus. The patient recovered well (normal left arm strength and range of motion and intact sensation to gross touch) and was discharged home on postoperative Day 5 on aspirin and coumadin with a goal international normalization ratio (INR) of two to three.

## Discussion

The clinical burden of peripheral arterial events is substantial, with high case fatality, poor functional outcome, and a high rate of emergency and subsequent vascular intervention to prevent death and limb loss [1]. We present a case of left upper extremity paresis secondary to acute brachial artery occlusion in a patient with active NSTEMI in the setting of paroxysmal atrial fibrillation. The patient was initially suspected to have a cerebrovascular attack (CVA); however, the CT head was negative for acute stroke. The presence of skin mottling, coolness of the arm, pallor, decreased sensation, and pulseless led to a further investigation with CTA of the upper extremity, confirming the diagnosis of acute left brachial artery occlusion.

Vascular disease is the leading cause of death and disability worldwide [2]. Acute limb ischemia (ALI) is the sudden decrease in limb perfusion, usually producing new or worsening symptoms and signs, and often threatening to limb [3]. ALI is considered a vascular emergency [4]. The incidence of ALI is 10 to 14 per 100000 per year [1,5]. The incidence of peripheral arterial events is similar between men and women, although women tend to have events at an older age [1]. The risks factors for atherosclerosis (hypertension, male sex, smoking, diabetes mellitus, and hyperlipidemia) are present in about 39.8% of patients with ALI. Previous atrial fibrillation was common in patients with ALI with a prevalence of 38.7% [1]. The most prevalent cause of ALI events was embolism 46.2% [1].

Patients with upper extremity ALI are more likely to have atrial fibrillation (50%) as compared to the 29.8% of patients with lower extremity acute limb ischemia [6], while patients with lower extremity ALI had a higher percentage of aortic or mitral valvular disease or intracardiac thrombus [6]. Upper extremity emboli are more frequent in women and patients with atrial fibrillation. Lower extremity emboli are more frequent in the presence of valvular disease or intracardiac thrombus and are associated with increased 30-day limb loss and mortality [6].

Upper extremity arterial occlusions can have a variety of presenting factors. Two of the most commonly described features are coldness and pain in the affected extremity, often occurring distally first and moving proximally. The pain is often a rest pain in acute occlusion but could also be claudication. Other presenting findings include paresthesia, paralysis, cyanosis, or pallor, decreased or absent radial or ulnar pulse, and gangrene of the digits [7]. If any of these symptoms are present, it is important to consider an arterial occlusion of the upper extremity. The patient will often also have predisposing conditions to thrombus formation and subsequent embolization. These conditions include myocardial infarction, atrial fibrillation, valvular heart disease, and other less common cardiac and noncardiac causes of emboli [7-9].

Clinical evaluation of patients with suspected arterial occlusion will demonstrate a change in temperature (coolness to touch) along the extremity affected as compared to the unaffected extremity. Furthermore, the bilateral pulse exam will demonstrate a diminished or absent palpable or Dopplerable pulse distal to the site of occlusion in the affected extremity. A CTA can be used to confirm the occlusion, while an urgent vascular surgery or an interventional radiology consult is necessary [4].

The three main treatments for acute limb ischemia include open surgical revascularization, endovascular revascularization, and anticoagulation with observation. Fluid resuscitation, pain control, and unfractionated heparin can be utilized to temporize the patient until definitive management can occur [4]. In patients presenting with ALI, the endovascular therapy and surgical revascularization approaches have similar rates of short-term and 12-month mortality, limb amputation, and recurrent ischemia [10].

## Conclusions

In evaluating a patient with concern for acute stroke with atypical presentation, it is essential to obtain a complete history and perform a rapid and thorough examination. Acute limb ischemia should be considered in the differential diagnosis of CVA with atypical presentation.
